# Overview of the Journal of Yeungnam Medical Science in 2025: submission trends, journal metrics, and appreciation for peer reviewers

**DOI:** 10.12701/jyms.2026.43.16

**Published:** 2026-01-29

**Authors:** Tae Gon Kim

**Affiliations:** Department of Plastic and Reconstructive Surgery, Yeungnam University College of Medicine, Daegu, Korea


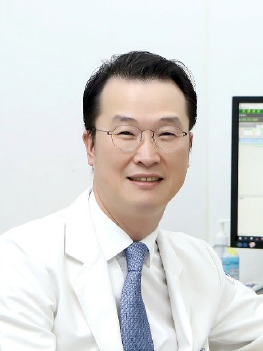


On behalf of the authors, readers, and editorial board of the *Journal of Yeungnam Medical Science* (JYMS), we would like to express our sincere gratitude to all peer reviewers who devoted their time and expertise to the journal throughout 2025. This editorial summarizes the growth and major developments of JYMS over the past year, including submission and publication trends, key journal metrics, and major editorial initiatives, and extends our appreciation to the reviewers who played a central role in maintaining the quality and integrity of the journal through the peer review process [1-4].

## Annual overview of journal performance in 2025

Since adopting the title *Journal of Yeungnam Medical Science* in 2022, JYMS has steadily strengthened its identity as an international medical journal. In 2023, the journal achieved indexing in both Scopus and the Emerging Sources Citation Index (ESCI), substantially increasing its global visibility and accessibility [1,2]. These milestones laid the foundation for subsequent growth in manuscript submissions and citation performance.

In 2025, JYMS further advanced its editorial policy by adopting the Continuous Article Publishing (CAP) model, enabling the prompt publication of accepted articles without waiting for issue compilation. This transition was implemented to facilitate the timely dissemination of research findings and improve overall editorial efficiency.

## Trends in manuscript submissions

Manuscript submissions to JYMS have shown a consistent upward trend over the past 4 years. The total number of submissions increased from 136 in 2022 to 246 in 2024 and reached 417 in 2025. Submissions from international authors increased markedly during this period, increasing from 55 in 2022 to 330 in 2025 (Table 1). This growth reflects the expanding international recognition and accessibility of JYMS.

Simultaneously, the acceptance rate gradually decreased as the submission volume increased and editorial standards were maintained. The overall acceptance rate declined from 46.3% in 2022 to 21.4% in 2024 and reached 16.5% in 2025 (Table 1). This trend indicates JYMS’s continued emphasis on scientific rigor and publication quality, alongside quantitative growth.

## Editorial efficiency after adoption of the Continuous Article Publishing model

Following its transition to the CAP model in 2025, JYMS achieved substantial improvements in editorial and publication efficiency. Compared with 2024, the overall timeline from submission to publication was substantially reduced.

**Fig. 1.**
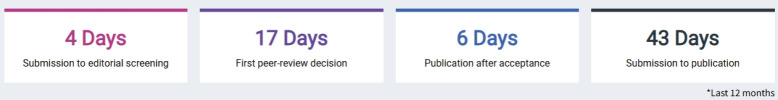
Timeline from manuscript submission to publication in the *Journal of Yeungnam Medical Science* over the last 12 months (https://e-jyms.org; accessed January 19, 2026).

On average, the time from submission to initial editorial screening was 4 days, the time to the first peer-review decision was 17 days, and the interval between acceptance and publication was 6 days. Consequently, the mean duration from submission to publication was reduced to 43 days (Fig. 1). These improvements represent a meaningful enhancement in editorial workflow and better address authors’ expectations for rapid publication.

## Introduction of a new article type: Medical student education section

In 2025, JYMS introduced a new article type, the “Medical student education section,” to promote scholarly engagement in undergraduate medical education. This section features educational case reports authored by medical students under the supervision of teaching physicians, with an emphasis on clinical reasoning and learning experience. These manuscripts follow the CAse REports (CARE) guidelines and include structured educational elements designed to enhance instructional value.

## Distribution of published articles by author countries

**Fig. 2.**
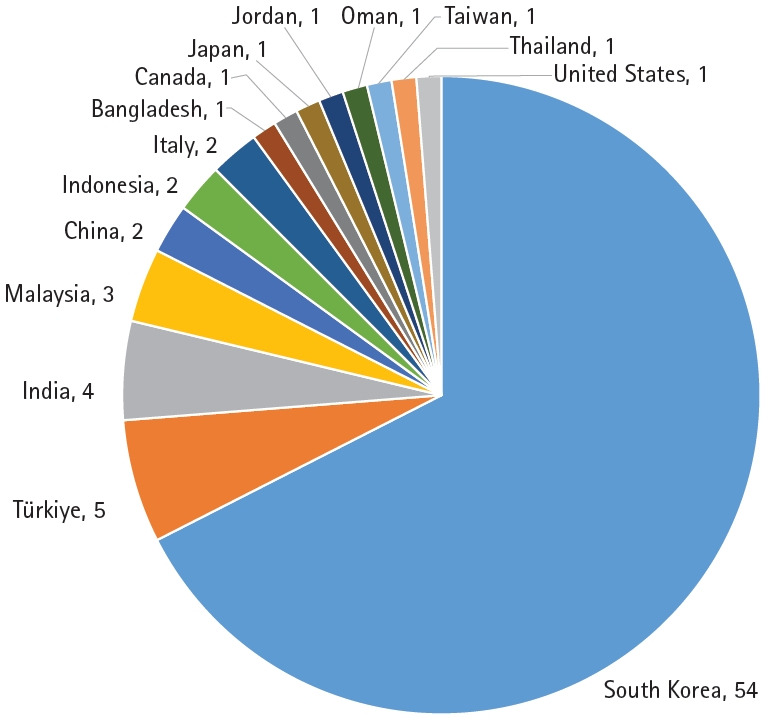
Number of articles published in the *Journal of Yeungnam Medical Science* in 2025 according to author countries.

The distribution of articles published in 2025 according to the authors’ countries is presented in Fig. 2. South Korea accounted for the largest proportion of published articles (54 articles, 67.5%), followed by Türkiye (five, 6.3%), India (four, 5.0%), Malaysia (three, 3.8%), China (two, 2.5%), Indonesia (two, 2.5%), Italy (two, 2.5%), and eight other countries (10%). These data demonstrate that JYMS continues to serve as a forum for international scholarly exchange while maintaining strong contributions from local researchers.

## Journal metrics and citation performance

**Fig. 3.**
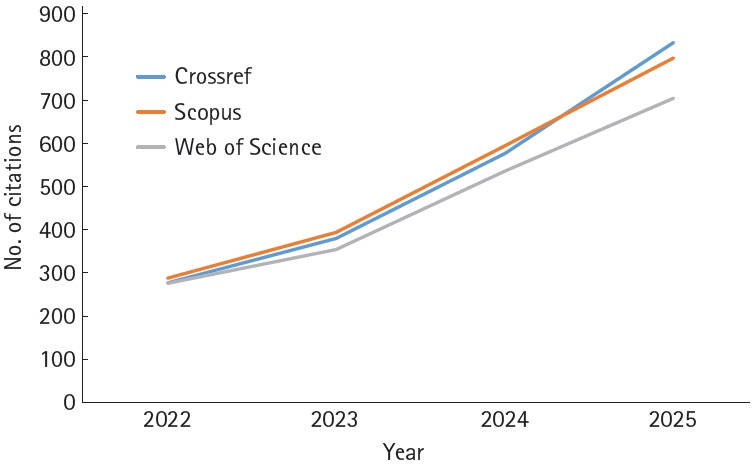
Number of citations of articles published in the *Journal of Yeungnam Medical Science* in Crossref Metadata, Scopus, and Web of Science Core Collection from 2022 to 2025 (calculated on December 30, 2025).

The citation trends for JYMS are shown in Fig. 3, illustrating a steady increase in citations across Crossref Metadata, Scopus, and the Web of Science Core Collection (Supplementary Table 1).

According to Journal Citation Reports (JCR) 2024, the Journal Impact Factor (JIF) of JYMS increased from 1.0 to 1.4, placing the journal in the second quartile (Q2) of the Medicine, General & Internal category. Similarly, the Scopus CiteScore 2024 rose from 0.8 to 2.0, corresponding to Q2 in the Multidisciplinary category [2,3]. Based on recent citation trends, the JIF and CiteScore are expected to approach approximately 2.0 and 3.3, respectively, in the 2026 evaluations.

## Access statistics and global readership

**Fig. 4.**
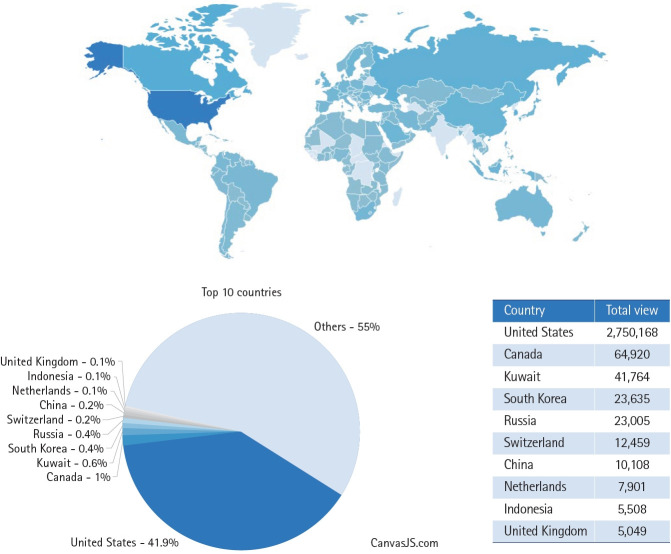
Top 10 countries with access to the *Journal of Yeungnam Medical Science* in 2025 (https://e-jyms.org/articles/metrics.php; accessed January 19, 2026).

The global visibility of JYMS is also reflected in the article access statistics. As shown in Fig. 4, articles published in JYMS in 2025 were accessed consistently by readers from various countries, indicating sustained international readership and broad journal accessibility.

## Copyright policy update

To further enhance the dissemination and reuse of published content, JYMS revised its copyright and open access policies in 2026. The journal transitioned from a Creative Commons Attribution-NonCommercial (CC BY-NC) license to a Creative Commons Attribution (CC BY) license. Under the CC BY license, JYMS articles may be used, shared, and reproduced for both noncommercial and commercial purposes, provided that proper attribution is given to the original work. This policy change is expected to encourage broader utilization of JYMS content and increase the academic impact of the journal.

## Appreciation for peer reviewers

In 2025, 177 reviewers from nine countries contributed to the peer review process of JYMS, as listed below. The editorial board expresses its deepest appreciation of their invaluable contributions throughout the year. We look forward to their continued support and collaboration as we advance through 2026.

**Canada**: Mathieu Boudier-Revéret (Centre hospitalier de l’Université de Montréal)

**China**: Li Tianxiao (Henan Provincial People’s Hospital)

**Czech Republic**: Mezian Kamal (Charles University)

**India**: Muralidharan Shrikanth (National Institute of Naturopathy, Ministry of Ayush), Santanu Mukhopadhyay (Malda Medical College and Hospital), Govindharaj Pitchaimani (Sri Ramachandra Institute of Higher Education and Research)

**Italy**: Ricci Vincenzo (Luigi Sacco University Hospital), Massimiliano Polastri (IRCCS Azienda Ospedaliero-Universitaria di Bologna)

**Malaysia**: Ramesh Kumaresan (AIMST University Malaysia), Cheng Lim Yin (Universiti Malaya Medical Centre)

**Taiwan**: Ming-Yen Hsiao, Ke-Vin Chang (National Taiwan University)

**Türkiye**: Amin Daemi (Çukurova University), Berkay Yalçınkaya (Hacettepe University Medical School)

**South Korea**: Pyunggoo Cho, Won-Tae Cho (Ajou University); Jun Lee, Jun-Won Seo (Chosun University); Seung Hoon Lee, In Jun Yang (Chungnam National University); Han Joon Bae, Hee Kyung Cho, Yoon Young Cho, Sang Gyu Gwak, Tae Chang Jang, Eon-Ju Jeon, Jonghae Kim, Suk-Bong Koh, Sang Kyu Kwak, Jae Hoon Lee, Jung A Lim, Ki Hyuk Park, Hun Mo Ryoo, Eunkyoung Shin, Sung-Doo Won (Daegu Catholic University); Min Young Lee (Daegu Fatima Hospital); Kyu Hwan Park (Daegu Veterans Hospital); Oh-Bin Kwon, Sung Suk Oh (Daegu-Gyeongbuk Medical Innovation Foundation); Sung-Man Bae (Dankook University); Seungwoo Lee (Dongguk University); Yun Woo Cho (Dr. Ahn’s Rehabilitation Clinic); Hye Jin Park (Eulji University); Young Hwii Ko, Seoyon Yang, You Gyoung Yi (Ewha Womans University); Jae-Kwan Lee (Gangneung-Wonju National University); Sun Hur, Soo Young Kim (Hallym University); Jong Ryeol Eun, Hyun-Min Seo (Hanyang University); Sangzin Ahn, Dongwook Lee (Inha University); Sung Hyuk Moon, June Namgung, Tae-Hoon No (Inje University); Jin Gwack, Gi Wook Kim, Ju-Hyung Lee (Jeonbuk National University); Su Min Lee (Kangwon National University); Jun Chul Byun, Yun-kyeong Cho, Jang Hyuk Cho, Eun Yeong Ha, Jin-Woong Jung, Sung Ae Kim, Hee Cheol Kim, Jin Kyung Kim, Sohyeon Kim, Younghwan Kim, Byoungje Kim, Nayeong Kong, Sae Min Kwon, Sang-Hun Lee, Ji Hoon Park, Kibeom Park (Keimyung University); Jae-Myung Kim, Min-Kyu Kim (Konyang University); Jong-Cheol Rah (Korea Brain Research Institute); Jae-Woo Cho, Wonseok Choi, Young Beom Seo (Korea University); Ku Sang Kim, Hwan Ho Lee, Young Lim Oh, Seol Hwa Seong, Ho Sik Shin (Kosin University); In-Kyung Jeong, Miae Oh (Kyung Hee University); Jae Yun Ahn, So-Hyun Bae, Eun Kyung Choi, Jae-Wook Chung, Yun Sok Ha, Jiyoon Jung, Yoonsuk Jung, Jong-Yeon Kim, Hae-Jin Ko, In Hee Lee, Sungbae Moon, Byung Geon Park, Jong Min Park, Hyun Wook Ryoo, Jinseok Yeo (Kyungpook National University); Chulyong Park (Nazareth General Hospital); Keun Jung Ryu (PMC Park Hospital); Lee Hwangbo, Jae-Hoon Kim, Hyeon Jeong Lee, Jae Min Lee, Su Bum Park (Pusan National University); Ju Sun Oh (Seoul Medical Center); Jinmyoung Dan, Yong Hee Hong, Hyung Mo Sung (Soonchunhyang University); Byung Joo Lee, Ji-Yun Park (University of Ulsan); Bong Gyu Choi, Hyun Jae Nam, Sang-Hyun Woo (W Hospital); Jung Min Bae, Sangwoon Bae, Young Kyung Bae, Eun Kyung Choi, Hyoung Chul Choi, Kang Un Choi, Joo Hyang Chun, Jun Young Do, Mi Jin Gu, Yejin Han, Jong Geol Jang, Ikchan Jeon, Jin Hee Jung, Seung Pil Jung, Ji Hoon Kang, Seok Hui Kang, So Hee Kang, A-Young Kim, Hong-Ju Kim, Hyun Je Kim, Jeong Kun Kim, Minchong Kim, Sang Won Kim, Sohyun Kim, Sung Bum Kim, Ung Kim, Yu Ra Kim, Zehwan Kim, Sung Ae Koh, Bon-Hoon Koo, So Young Kwak, Jin-Ju Kwon, Chan-Hee Lee, Chu Hee Lee, Jang Hoon Lee, Keun-Mi Lee, Chul Hyun Park, Hosun Park, Il Rae Park, Jae Won Park, Jaehyeon Park, Jong Il Park, Jong Soo Park, Wook Tae Park, Young-Nam Roh, Wan Seok Seo, Kyeong Cheol Shin, Jang Won Son, Chang Hoon Woo, Ji Sung Yoon, Seokho Yun (Yeungnam University); Jung Ho Kim, Seung Woo Kim, Eun Byoul Lee, Kyung Hye Park, Moo Suk Park, Dong Ah Shin (Yonsei University).

## Figures and Tables

**Table 1.** Trends in manuscript submissions and acceptance rates by author origin and overall acceptance rate (2022–2025)

Category	2022	2023	2024	2025
Local	81	99	72	87
International	55	108	174	330
Total	136	207	246	417
Acceptance rate (%)	46.3	28.3	21.4	16.5

## Supplementary materials

Supplementary Table can be found at https://doi.org/10.12701/jyms.2026.43.16.

Supplementary Table 1. Number of citations of articles published in the *Journal of Yeungnam Medical Science* indexed in Crossref Metadata, Scopus, and Web of Science Core Collection (2022–2025)
