# Supplementary material for: Overview of the Journal of Yeungnam Medical Science in 2025: submission trends, journal metrics, and appreciation for peer reviewers
Source: J Yeungnam Med Sci. 2026 Jan 29:jyms.2026.43.16. doi: 10.12701/jyms.2026.43.16 (PMC12957858; doi:10.12701/jyms.2026.43.16)
Supplement: Supplementary Table 1. — Number of citations of articles published in the Journal of Yeungnam Medical Science indexed in Crossref Metadata, Scopus, and Web of Science Core Collection (2022–2025) [file jyms-2026-43-16-Supplementary-Table-1.pdf]

Supplementary Table 1. Number of citations of articles published in the Journal of Yeungnam Medical Science indexed in Crossref Metadata, Scopus, and Web of Science Core Collection (2022–2025)

Year	Crossref	Scopus	Web of Science
2022	279	290	278
2023	383	397	357
2024	581	599	540
2025	840	804	710

Data calculated on December 30, 2025.
